# Relationship Between Outdoor Air Pollutant Exposure and Premature Delivery in China- Systematic Review and Meta-Analysis

**DOI:** 10.3389/ijph.2023.1606226

**Published:** 2023-10-09

**Authors:** Xue Wang, Xin Wang, Chenghua Gao, Xiaoqian Xu, Lehui Li, Yan Liu, Zichao Li, Yuan Xia, Xin Fang

**Affiliations:** ^1^ School of Public Health of Inner Mongolia Medical University, Hohhot, China; ^2^ Division of Molecular Signaling, Department of the Advanced Biomedical Research, Interdisciplinary Graduate School of Medicine, University of Yamanashi, Kofu, Japan

**Keywords:** atmospheric pollutants, preterm birth, meta-analysis, China, adverse pregnancy outcome

## Abstract

**Objective:** Preterm birth (PTB) is considered as a public health problem and one of the main risk factors related to the global disease burden. The purpose of this study aims to explore the influence of exposure to major air pollutants at different pregnancies on PTB.

**Methods:** The relationship between air pollutants and PTB in China was collected from cohort studies and case-control studies published before 30 April 2022. Meta-analysis was carried out with STATA 15.0 software.

**Results:** A total of 2,115 papers were retrieved, of which 18 papers met the inclusion criteria. The comprehensive effect of pollutant exposure and PTB were calculated. PM_2.5_ during entire pregnancy and O_3_ exposure during third trimester were positively associated with preterm birth. Every 10 μg/m^3^ increase in the average concentration of PM_2.5_ during the whole pregnancy will increase the risk of premature delivery by 4%, and every 10 μg/m^3^ increase in the average concentration of O_3_ in the third trimester will increase the risk of premature delivery by 1%.

**Conclusion:** Exposure to PM_2.5_ entire prenatal pregnancy and O_3_ in third trimester is associated with an increased risk of preterm birth occurrence.

## Introduction

Preterm birth (PTB) is defined as delivery before 37 weeks of pregnancy, which has long-term negative impacts on the development of newborns and may lead to stunting. PTB is becoming more and more common in developing and even some developed countries [1], although the underlying causes are still unknown. Premature delivery is a common adverse pregnancy outcome. Because of its serious impact on life and health, it brings a great burden on families and society, leading to perinatal and early neonatal death (especially in developing countries) and immature organ development, which leads to physical disability or mental disorders [2, 3]. PTB is regarded as a public health issue and one of the key risk factors contributing to the Global Burden of Disease. As a result, there is an urgent need to investigate PTB risk factors in order to reduce the occurrence of PTB and thus improve human health.

PTB has been linked to a variety of factors, including social and economic status, race, genetic influences, medical conditions, mental disorders, and environmental exposures [4]. Air pollution is a major component of environmental pollution, and it is considered as a risk factor of many diseases. According to the latest estimate of the World Health Organization, about 90% of the world’s population breathes air with high concentrations of pollutants, and as many as 7 million people die every year due to environmental and indoor air pollution [5]. The impact of air pollution on health has become a global public health problem (6). In addition to causing respiratory [7, 8] and cardiovascular diseases [9], more and more studies have investigated the influence of air pollution on premature delivery, including China [10], South Korea [11], Japan [12] and the United States [13]. Although most published studies have reported that various air pollutants are related to PTB, the nature of the pollutants studied and the related pregnancy outcomes are different. At present, there is a lack of toxicological data to guide pregnant women to choose the most relevant vulnerable exposure window [14]. Previous surveys have used a wide range of exposure windows (i.e., weeks, months and 3 months) to determine the time relationship between air pollution and adverse pregnancy outcomes [15]. Some studies have reported that exposure in the first trimester of pregnancy is associated with an increased risk of PTB [16]. Other studies have shown that exposure in the third trimester has a greater impact [17]. Therefore, the relationship between different pollutants exposure and different adverse pregnancy outcomes is complicated, and different periods of pollutant exposure during pregnancy have different effects on pregnancy outcomes [18]. Some previous studies have discussed the effect of air pollution on PTB [19–21], but they are mainly carried out in developed countries. Although the level of air pollution in Asian countries is usually higher, there has been a lack of research in these countries [10, 22]. Fetuses are particularly sensitive to environmental hazards because cell proliferation, differentiation and organ development accelerate during pregnancy, which may directly damage the fetus or interfere with placental function [23]. Several possible biological approaches have been put forward in previous studies. On the one hand, placental DNA adducts produced by air pollution are more common in mothers exposed to high levels of air pollution [24]. The high expression of fetal DNA adducts may reduce the ability of DNA repair and detoxification enzymes [25]. In previous studies, DNA adducts were associated with shortened pregnancy [26]. On the other hand, its mechanism may be related to hematological factors. The increase of blood viscosity caused by exposure to air pollution and hematological factors may be related to insufficient placental perfusion, which leads to PTB(24). Animal studies also support the finding that exposure to urban air pollution during pregnancy will inhibit fetal growth, which is closely related to the risk of premature delivery [27]. Therefore, this paper plans to collect the previous cohort and case-control studies, effectively increasing the sample size, and further improving the evidence level by including only the studies with high Newcastle-Ottawa Quality Score (NOS) and discussing the effects of air pollution exposure during different pregnancies on PTB.

## Methods

### Literature Search

Inclusion criteria: 1) published before 31 April 2022, the contents of the literature were all independent epidemiological findings; 2) the study was about the effect of atmospheric pollutants on preterm birth in China; 3) all studies defined delivery at less than 37 weeks of gestational age as preterm birth according to WHO criteria; and defined the 3 gestational window periods in the following way, namely: the first 3 months of pregnancy (before the end of 12 weeks of gestation) as early gestation, second trimester at the end of 13–27 weeks of gestation, and third trimester after 28 weeks of gestation; 4) the risk factors studied included air pollutants; 5) the results of each literature is the quantitative dose-response relationships between air pollution and premature delivery, including parameter estimation and 95% CI of the risk of premature delivery with the increase in air pollutant concentration of 10 μg/m^3^; 6) the study methods were cohort studies or case-control studies.

Exclusion criteria: 1) animal studies, *in vitro* toxicology studies, reviews and review articles; 2) studies with risk factors limited to indoor pollution, some kind of heavy metal or organic pollution; 3) studies examining the effects of air pollution due to occupational exposure on adverse pregnancy outcomes; 4) studies on the birth outcomes except premature delivery; 5) studies for which RR values and 95% CI were not available; 6) the original article contains no original data.

### Literature Search Strategy

The literature on the effect of outdoor air pollution on preterm birth in China published at home and abroad before 31 April 2022, was collected by computer search of PubMed, Cochrane Library, Web of science, China Journal Full Text Database (CNKI), Wanfang Science and Technology Information Database and Weipu Chinese Science and Technology Journal Full Text Database, and by tracing the relevant literature. The retrieved keywords were: air pollution, environmental pollution, air quality, atmospheric pollution, atmospheric pollutants, PM_10_, PM_2.5_, NO_2_, SO_2_, O_3_, sulfur dioxide, nitrogen dioxide, ozone, particulate matter, adverse birth outcomes, adverse pregnancy outcomes, preterm birth premature birth, preterm delivery, small for gestational age, Chinese, China, atmospheric pollution, air pollution, particulate matter, respirable particulate matter, fine particulate matter, sulfur dioxide, nitrogen dioxide, ozone, adverse pregnancy outcomes, preterm birth, less than gestational age.

### Literature Screening and Extraction

The literature search was conducted independently by two researchers, and the literature initially examined was screened one by one according to the inclusion and exclusion criteria, and relevant information was extracted and checked for differences. If they had different opinions, they all discussed and unified. The information extracted included authors, year of publication, study site, study duration, number of study participants, study method, exposure assessment method, type of air pollutant, and period of exposure. Because the sensitivity of fetuses to pollutant exposure in different periods is different, and the results of the influence of pollutant exposure on premature delivery in different periods may be different, most of the original documents are analyzed separately according to gestational period, so the data are extracted separately according to gestational period.

### Statistical Analysis

Statistical software Stata 15.0 was used for meta-analysis of data, and *I* ^*2*^ statistics were used to measure the heterogeneity of individual research results. If there was no heterogeneity between the studies (*I* ^*2*^ < 50% or *p* > 0.10), the fixed effect model was used to calculate the comprehensive RR value and its 95% CI. If there is heterogeneity, a random effects model was used and subgroup analysis was used to explore the source of heterogeneity. Sensitivity analysis was used to evaluate the stability of the Meta-analysis findings and to determine the differences in the combined effect values before and after excluding a particular study. Egger linear regression analysis was used to analyze the publication bias, and the test level was bilateral α = 0.01.

## Results

### General Description

**FIGURE 1:**
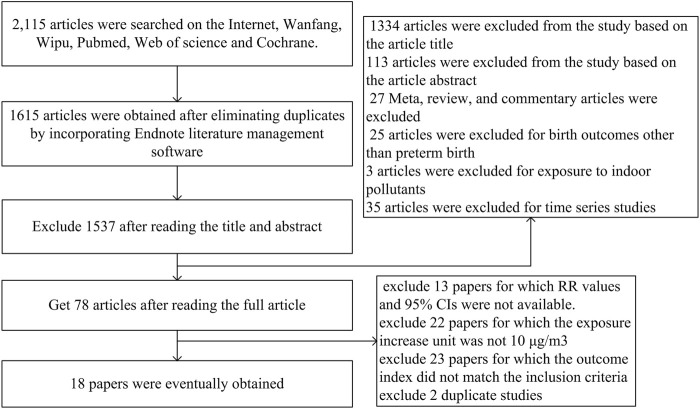
Flow chart of literature screening (China, 2023).

A total of 1,615 researches were retrieved. After a preliminary screening of the title and abstract, 78 potentially suitable studies were selected. Figure 1 depicts the flowchart of the study search and selection in the Meta-analysis and lists the 18 publications that were deemed eligible after additional assessment of the complete texts of these articles. The data features of the 18 papers included in this meta-analysis are shown in Table 1. In these 18 investigations, 11, 8, 4, 6 and 3 investigated the effects of PM_2.5_, PM_10_, SO_2_, NO_2_ and O_3_ on PTB respectively. Only two surveys have been conducted on Chinese national populations, most of which (*n* = 11) were conducted in southern cities, followed by northern cities (*n* = 5). There were about 1,882–1,240,978 participants in these studies. Three researchers used satellite data, and 15 researchers used monitoring station data to determine exposure assessment methods.

**TABLE 1:** Basic information of the literature included in the Meta-analysis. (China, 2023).

Authors	Publication date	Study site	Study years	Sample size	Pollutant	Study design	Exposure assessment	NOS score
[34]	2015	Beijing	2007–2010	50,874	PM_10_, NO_2_	Case control study	Monitoring station	6
[35]	2016	Taiyuan	2013–2014	1,882	PM_2.5_	Case control study	Monitoring station	6
[36]	2015	Wuhan	2010–2013	95,911	O_3_	Case control study	Monitoring station	6
[37]	2018	China	2013–2014	1,240,978	PM_2.5_, PM_10_	cohort study	satellite remote sensing	8
[38]	2018	Taizhou	2013–2016	24,246	PM_2.5_, PM_10_, NO_2_	cohort study	Monitoring station	7
[39]	2018	Shanghai	2011–2014	132,783	PM_2.5_	Case control study	satellite remote sensing	6
[40]	2018	Lanzhou	2010–2012	8,969	PM_10_	Case control study	Monitoring station	8
[41]	2019	Shanghai	2014–2015	25,493	NO_2_	Case control study	Monitoring station	5
[42]	2019	Guangdong	2014–2017	1,455,026	PM_2.5_	Case control study	Monitoring station	6
[43]	2022	Henan	2013–2018	196,780	PM_2.5_	cohort study	Monitoring station	7
[44]	2019	Guangdong	2014–2015	3,550	PM_2.5_, PM_10_, SO_2_, NO_2_, O_3_	Case control study	Monitoring station	5
[45]	2020	Shanghai	2013–2016	3,692	PM_2.5_	Case control study	satellite remote sensing	5
[46]	2019	Guangdong	2014–2017	1,455,026	O_3_	cohort study	Monitoring station	7
[47]	2021	Shiyan	2015–2017	13,111	PM_10_, PM_2.5_, NO_2_, SO_2_	cohort study	Monitoring station	7
[48]	2021	China	2009–2011	5,976	PM_2.5_	cohort study	Monitoring station	7
[49]	2022	Yan’ an	2018–2019	10,722	PM_2.5_, PM_10_, SO_2_, NO_2_, O_3_	Case control study	Monitoring station	6
[50]	2022	Shanghai	2016.1–2016.6	10,370	PM_2.5_	Case control study	Monitoring station	5
[51]	2022	Chongqing	2015–2020	572,116	PM_2.5_, PM_10_, SO_2_, O_3_, NO_2_	cohort study	Monitoring station	7

### Heterogeneity Test

**FIGURE 2:**
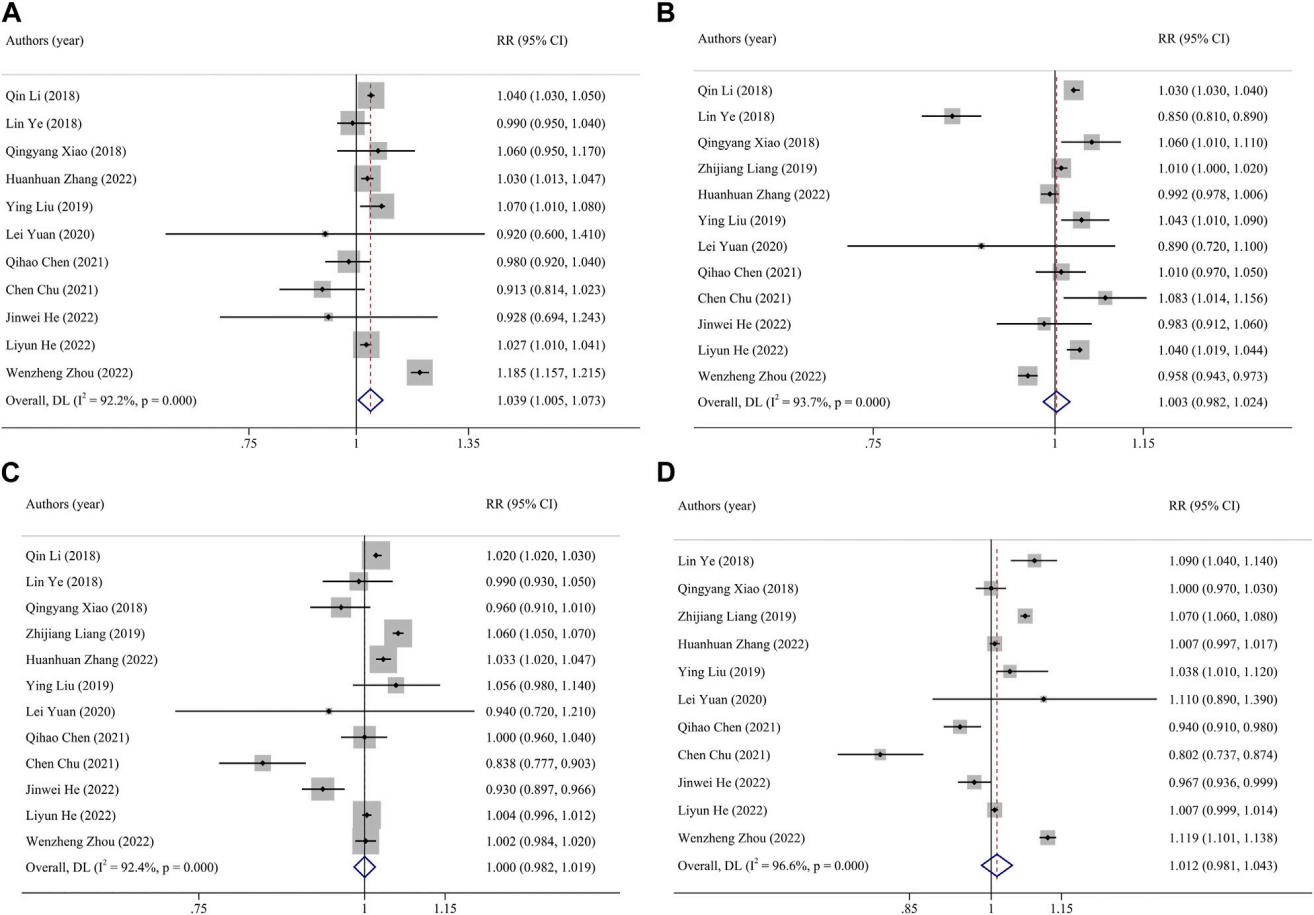
Funnel plot of the association between exposure to PM_2.5_ and premature birth. Pooled estimates of effect size are indicated by vertical points of diamonds, size of shaded area around the diamond is proportional to weight, and 95% *CI* are represented by horizontal line. (**(A)**, entire pregnancy; **(B)**, first trimester; **(C)**, second trimester; **(D)**, third trimester). (China, 2023).

The results of exposure to PM_2.5_, PM_10_, SO_2_, NO_2_ and O_3_ in different pregnancy were analyzed by heterogeneity. The heterogeneity of O_3_ exposure results in the third trimester of pregnancy was low, and the effect is estimated by using fixed effect model, while the results of the other pollutants had high heterogeneity, the random effect model is used to the research results. The results showed that every 10 μg/m^3^ increase in PM_2.5_ concentration during the whole pregnancy increases the risk of PTB by 4% (RR: 1.04, 95% CI: 1.01, 1.07). The risk of PTB increased by 1% (RR: 1.01, 95% CI: 1.01, 1.02) for every 10 μg/m^3^ increase in O_3_ concentration in the third trimester. The effects of PM_10_, SO_2_ and NO_2_ exposure in different pregnancy on PTB were not found statistically significant. Table 2 and Figure 2 (Supplementary Figures S1–S3).

**TABLE 2:** Summary of integrated impact estimates of premature birth associated with air pollution (PM_2.5_, PM_10_, NO_2_, SO_2_ and O_3_) (China, 2023).

Air pollution and PTB	Trimester	No. of studies	Test of association		Test of heterogeneity		Egger’s test
			*RR*	95% *CI*	*I* ^2^ (%)	*p*-Value	*p*-Value
PM_2.5_	Entire	10	1.04	1.01, 1.07	91.5	<0.001	0.916
	First	11	1.00	0.97, 1.02	94.3	<0.001	0.209
	Second	11	1.00	0.97, 1.02	92.4	<0.001	0.208
	Third	10	1.01	0.97, 1.05	96.5	<0.001	0.453
PM_10_	Entire	7	1.03	0.99, 1.06	91.4	<0.001	0.321
	First	7	0.97	0.95, 1.00	84.2	<0.001	0.559
	Second	8	1.00	0.97, 1.02	72.0	0.001	0.546
	Third	7	1.03	0.98, 1.08	94.6	<0.001	0.506
SO_2_	Entire	4	0.95	0.69, 1.00	63.9	0.040	0.978
	First	4	0.92	0.72, 1.13	98.3	<0.001	0.851
	Second	4	0.96	0.80, 1.10	96.1	<0.001	0.761
	Third	4	0.96	0.85, 1.06	92.0	<0.001	0.462
NO_2_	Entire	5	1.06	0.92, 1.20	94.2	<0.001	0.181
	First	6	0.97	0.93, 1.01	67.7	0.009	0.041
	Second	6	1.03	0.94, 1.11	91.7	<0.001	0.060
	Third	6	1.00	0.94, 1.06	89.4	<0.001	0.213
O_3_	Entire	4	0.98	0.91, 1.05	98.2	<0.001	0.898
	First	4	0.98	0.95, 1.02	97.8	<0.001	0.330
	Second	4	0.98	0.93, 1.02	98.6	<0.001	0.449
	Third	4	1.01	1.01, 1.02	0.0	0.712	0.593

### Subgroup Analysis

**FIGURE 3:**
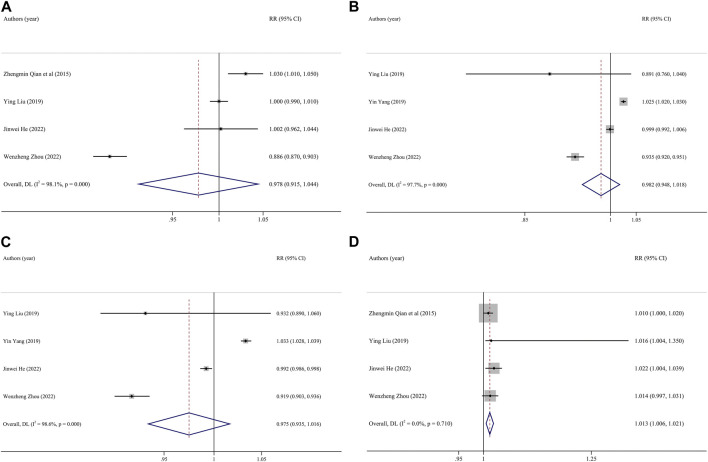
Funnel plot of the association between exposure to O_3_ and premature birth. Pooled estimates of effect size are indicated by vertical points of diamonds, size of shaded area around the diamond is proportional to weight, and 95% *CI* are represented by horizontal line. (**(A)**, entire pregnancy; **(B)** first trimester; **(C)**, second trimester; **(D)**, third trimester). (China, 2023).

By further analysis of different research methods (cohort and case-control study). In the case-control study, every 10 μg/m^3^ increase in PM_2.5_ concentration during the whole pregnancy increased the risk of PTB by 4% (RR:1.04, 95% CI: 1.01, 1.07). Every 10 μg/m^3^ increase in the average concentration of SO_2_ in early and late pregnancy will increase the risk of PTB by 4% (first pregnancy RR: 1.04, 95% CI: 1.02, 1.07, late pregnancy RR: 1.04, 95% CI: 1.01, 1.06). In the cohort study, the risk of PTB was reduced by 11%, 15%, and 12% for each 10 μg/m^3^ increase in SO_2_ concentration throughout pregnancy, mid-term and late in pregnancy (whole pregnancy RR: 0.89, 95% CI: 0.84, 0.94, second pregnancy RR: 0.85, 95% CI: 0.74, 0.97, late pregnancy RR: 0.88, 95% CI: 0.82). The risk of PTB was reduced by 6% for each 10 μg/m^3^ increase in NO_2_ concentration in first pregnancy (RR: 0.94, 95% CI: 0.90, 0.99). According to the study areas (southern cities, northern cities and the whole country), the influence of pollutant exposure on PTB was evaluated by stratified analysis. The results show that the influence of PM_2.5_ on PTB has statistical significance in the study of northern cities (RR: 1.03, 95% CI: 1.01, 1.05). Studies conducted in southern cities showed that the risk of PTB increased by 5% for every 10 μg/m^3^ increase in PM_10_ concentration during the whole pregnancy (RR: 1.05, 95% CI: 1.02, 1.09). Because the exposure concentration of pollutants may have different effects on health [28], we stratify the concentration of pollutants according to WHO standards. Stratified according to different concentrations of pollutants, when the concentration of PM_2.5_ exceeds 50 μg/m^3^, the PTB will increase by 2.5% (RR:1.025, 95% CI: 1.01, 1.04) for every 10 μg/m^3^ increase of PM_2.5_ during the whole pregnancy. 9% for every 10 μg/m^3^ increase of PM_2.5_ when the concentration is lower than 50 μg/m^3^. The heterogeneity of PM_2.5_ was significantly reduced in case-control study, northern city study and study with exposure concentration higher than 50 μg/m^3^. The heterogeneity of PM_10_ in southern cities, SO_2_ in cohort study and case-control study, and NO_2_ cohort study was significantly reduced. These may be the heterogeneous sources of merger effect. Table 3 and Figure 3.

**TABLE 3:** Subgroup analysis of premature birth associated with air pollution (PM_2.5_, PM_10,_ SO_2_, NO_2_ and O_3_) in different late gestational periods. (China, 2023).

Exposure	Subgroup	PM2.5		PM10	
		Division	RR (95% CI)	*n*	RR (95% CI)
Entire	Research type	cohort study *n* = 6	1.03 (0.98, 1.08)	*n* = 4	1.03 (0.97, 1.09)
		Case control study *n* = 5	1.04 (1.01, 1.07)^a^	*n* = 3	1.03 (1.00, 1.05)^a^
	region	north *n* = 2	1.03 (1.01, 1.05)^a^	*n* = 2	1.01 (0.96, 1.05)^a^
		south *n* = 7	1.05 (0.98, 1.12)	*n* = 4	1.05 (1.02, 1.09)
		China *n* = 2	0.99 (0.87, 1.11)	*n* = 1	1.00 (1.00, 1.00)^a^
	concentration	>50 μg/m^3^ *n* = 7	1.03 (1.01, 1.04)	>100 μg/m^3^ *n* = 3	1.01 (0.96, 1.07)
		<50 μg/m^3^ *n* = 5	1.09 (1.03, 1.17)	<100 μg/m^3^ *n* = 5	1.03 (0.99, 1.07)
First	Research type	cohort study *n* = 6	0.98 (0.94, 1.02)^a^	*n* = 3	0.93 (0.86, 1.01)
		Case control study *n* = 6	1.03 (1.00, 1.05)	*n* = 4	1.00 (0.99, 1.01)
	region	north *n* = 2	0.99 (0.98, 1.01)^a^	*n* = 3	1.00 (0.99, 1.01)
		south *n* = 7	0.99 (0.96, 1.03)	*n* = 4	0.96 (0.89, 1.02)
		China *n* = 2	1.04 (1.00, 1.09)	*n* = 0	
Second	Research type	cohort study *n* = 6	1.00 (0.97, 1.02)	*n* = 4	1.01 (0.97, 1.04)
		Case control study *n* = 6	1.00 (0.96, 1.04)	*n* = 4	1.00 (0.98, 1.02)^a^
	region	north *n* = 2	0.98 (0.88, 1.08)	*n* = 3	1.00 (0.99, 1.01)^a^
		south *n* = 7	1.01 (0.98, 1.04)	*n* = 4	0.96 (0.89, 1.02)
		China *n* = 2	0.93 (0.75, 1.11)	*n* = 1	1.01 (1.00, 1.01)^a^
Third	Research type	cohort study *n* = 5	1.00 (0.92, 1.07)	*n* = 3	1.04 (0.95, 1.08)
		Case control study *n* = 6	1.02 (0.98, 1.06)	*n* = 4	1.02 (1.00, 1.03)
	region	north *n* = 2	0.99 (0.95, 1.03)	*n* = 3	1.01 (1.00, 1.03)
		south *n* = 7	1.04 (1.00, 1.08)	*n* = 4	1.04 (0.97, 1.11)
		China *n* = 2	0.80 (0.73, 0.87)	*n* = 0	—

Exposure	Subgroup	SO_2_		NO_2_		O_3_	
		*n*	*RR* (95% *CI*)	*n*	*RR* (95% *CI*)	*n*	*RR* (95% *CI*)
Entire	Research type	cohort study *n* = 2	0.89 (0.84, 0.94)^a^	*n* = 3	1.07 (0.81, 1.33)	*n* = 1	0.89 (0.87, 0.90)^a^
		Case control study *n* = 2	0.99 (0.90, 1.08)	*n* = 2	1.05 (0.99, 1.11)^a^	*n* = 3	1.01 (0.99, 1.03)
	region	north *n* = 1	0.95 (0.93, 0.98)^a^	*n* = 1	1.01 (0.62, 1.40)^a^	*n* = 1	1.00 (0.96, 1.04)^a^
		south *n* = 3	0.94 (0.84, 1.05)	*n* = 4	1.06 (0.91, 1.22)	*n* = 3	0.97 (0.89, 1.05)
		China *n* = 0	—	*n* = 0	—	*n* = 0	—
	concentration	>50 μg/m^3^ *n* = 0	—	>50 μg/m^3^ *n* = 1	0.98 (0.94, 1.02)^a^	>120 μg/m^3^ *n* = 0	—
		<50 μg/m^3^ *n* = 4	0.95 (0.89, 1.01)	<50 μg/m^3^ *n* = 6	1.05 (0.93, 1.18)	<120 μg/m^3^ *n* = 5	0.99 (0.94, 1.04)
First	Research type	cohort study *n* = 2	0.83 (0.63, 1.03)	*n* = 3	0.94 (0.90, 0.99)^a^	*n* = 2	0.98 (0.89, 1.07)
		Case control study *n* = 2	1.04 (1.02, 1.07)^a^	*n* = 3	1.00 (0.92, 1.08)^a^	*n* = 2	0.97 (0.87, 1.06)
	region	north *n* = 1	1.05 (1.02, 1.07)^a^	*n* = 2	0.98 (0.94, 1.02)^a^	*n* = 1	1.00 (0.99, 1.01)^a^
		south *n* = 3	0.87 (0.70, 1.05)	*n* = 4	0.98 (0.91, 1.05)	*n* = 3	0.96 (0.88, 1.04)
		China *n* = 0	—	*n* = 0	—	*n* = 0	—
Second	Research type	cohort study n = 2	0.85 (0.74, 0.97)	*n* = 3	1.05 (0.86, 1.24)	*n* = 2	0.98 (0.86, 1.09)
		Case control study *n* = 2	1.02 (1.00, 1.05)^a^	*n* = 3	0.99 (0.93, 1.06)^a^	*n* = 2	0.98 (0.93, 1.03)^a^
	region	north *n* = 1	1.02 (1.00, 1.05)^a^	*n* = 2	0.97 (0.92, 1.01)^a^	*n* = 1	0.99 (0.99, 1.00)^a^
		south *n* = 3	0.91 (0.76, 1.06)	*n* = 4	1.05 (0.90, 1.21)	*n* = 3	0.96 (0.87, 1.06)
		China *n* = 0	—	*n* = 0	—	*n* = 0	—
Third	Research type	cohort study *n* = 2	0.88 (0.82, 0.94)^a^	*n* = 3	1.01 (0.87, 1.15)	*n* = 1	1.01 (1.00, 1.03)^a^
		Case control study *n* = 2	1.04 (1.01, 1.06)	*n* = 3	0.99 (0.96, 1.02)	*n* = 3	1.01 (1.00, 1.02)^a^
	region	north *n* = 1	1.03 (1.01, 1.06)	*n* = 2	0.99 (0.96, 1.02)	*n* = 1	1.02 (1.00, 1.04)^a^
		south *n* = 3	0.92 (0.81, 1.04)	*n* = 4	1.00 (0.89, 1.11)	*n* = 3	1.01 (1.00, 1.02)^a^
		China *n* = 0	—	*n* = 0	—	*n* = 0	—

^a^ *I* *^2^* < 50%.

### Publication Bias and Sensitivity Analysis

Taking lgRR as the abscissa and its standard deviation as the ordinate, the funnel diagram was drawn according to pregnancy periods, and it was found that there was no publication bias in the included literature. By excluding studies one by one in turn, the sensitivity analysis was conducted to evaluate the robustness of the effect, and it was found that the effect of various pollutants exposure on PTB risk were stable in this meta-analysis (Supplementary Figures S4–S25).

## Discussion

The influence of air pollutants on premature delivery has become the focus of attention. This Meta-analysis included 18 eligible studies to investigate the correlation between the effects of each 10 μg/m^3^ increase in the mean concentration of five major ambient air pollutants (including PM_2.5_, PM _10_, NO_2_, SO_2_ and O_3_) during pregnancy exposure on preterm birth. The results showed that exposure to PM_2.5_ throughout pregnancy and O_3_ in late pregnancy was positively correlated with PTB. Every 10 μg/m^3^ increase in the average concentration of PM_2.5_ in the whole pregnancy increased the risk of premature delivery by 4%, and every 10 μg/m^3^ increase in the average concentration of O_3_ in the third trimester increased the risk of premature delivery by 1%. Every increase of 10 μg/m^3^ in the exposure concentration of residual pollutants during different pregnancy has no significant effect on the incidence of premature delivery. As a major component of air pollution mixture, PM_2.5_ is more likely to harm human health due to its special characteristics, such as small size, large specific surface area, long floating time in the air, and adsorption capacity [29]. SUN, Chen et al. [29, 30] similarly found that PM_2.5_ exposure throughout pregnancy was associated with preterm delivery, and an increase of PM_2.5_ in early to mid-late pregnancy 10 μg/m^3^, the risk of preterm birth occurrence was not significant. Luo et al. [31] found that PM_2.5_ exposure throughout pregnancy was not associated with preterm birth. Liu et al. [32] reported results shows that there is a significant correlation between low exposure of PM_2.5_ in early pregnancy and premature delivery. This inconsistency may be due to the different number of studies involved due to different increments in pollutant exposure levels, search times, and search scopes, which may be the main reason for the discrepancy in study results. Current epidemiological evidence suggests that women in the first trimester of pregnancy tend to be less active outdoors when reported PM_2.5_ concentrations are high, which may overestimate ambient PM_2.5_ exposure and lead to bias. However, during periods of relatively low reported PM_2.5_ concentrations, they are relatively less aware of self-protection against air pollution, which may lead to a high risk of preterm birth.

Pregnancy is a time when people are most susceptible to the effects of air pollution, although past researches have produced mixed findings. Early gestation (first or third month), according to some studies, is the window of susceptibility, however later gestation is supported by other studies (third gestation, last month, or last week). In the present study, the effect of different gestational pollutant exposures on preterm birth was investigated and only O_3_ exposure in late pregnancy was found to promote preterm birth. The results of Ju et al. [33] also showed that O_3_ exposure in early and mid-late pregnancy was significantly associated with preterm birth. This is similar to our results, suggesting that the susceptibility window may be related to the specificity of different pollutants and needs to be further explored.

It is well known that the toxicity and health effects of air pollutants may vary by geographic region. Therefore, it is reasonable to assess the effects of different gestational air pollutant exposures on PTB by regional subgroup analysis. Subgroup analyses in different regions showed regional differences in the effects of air pollution on PTB. The results showed that the exposure of PM_2.5_ in the northern area and PM_10_ in southern area during pregnancy was significantly related to the increase of PTB, but other pollutants did not find this correlation. Due to the differences in living habits, climate and pollutant concentrations between the northern and southern regions, it was found that the concentration of PM_2.5_ in the northern region was higher than that in the southern region due to coal burning in winter. The quality of the results from long-term cohort studies is obviously better than that of case-control studies. Especially the prospective cohort studies, is considered to be the most reliable results in observational epidemiology. Therefore, we used different research methods to carry out subgroup analysis. The results showed that SO_2_ exposure in the second and third trimester of pregnancy was significantly correlated with the decrease of PTB, while in the case-control study, SO_2_ exposure in the first trimester of pregnancy was significantly correlated with the increase of PTB. The cohort study found that NO_2_ exposure in early pregnancy was significantly correlated with the decrease of PTB, while NO_2_ exposure in middle and late pregnancy was not significantly correlated with premature delivery.

We discovered substantial variability among the included studies, which is predictable in view of the limitations of our meta-analysis. In order to quantitatively aggregate individual estimates from studies with substantial heterogeneity, we employed a random effects model. Subgroup analysis was also used to investigate the causes of heterogeneity. The results show that, even though the study design and study area only partially explain the variability, most subgroup analyses still revealed significant heterogeneity. These results imply that due to the small number of related studies, variability of the included studies may be affected by other factors that are not considered in this analysis, such as socio-economic status and chemical composition of pollutants. Therefore, more studies are needed to investigate the causes of heterogeneity in the future. In several studies, personal samplers were used to measure the exposure of pregnant women to air pollutants, and the results were more accurate. Therefore, more research on personal information might be helpful for future analysis.

### Conclusion

Exposure to PM_2.5_ throughout pregnancy may be related to the increased risk of PTB, but prenatal exposure to PM_10_, SO_2_, NO_2_ and O_3_ has no significant correlation. Analysis stratified by different gestational periods showed that O_3_ exposure in late pregnancy may increase the risk of preterm birth occurrence, but the overall level of effect was not high. The sensitivity windows for different pollutants are different, suggesting that more in-depth research is needed in this field in the future.
