# Supplementary material for: Relationship Between Outdoor Air Pollutant Exposure and Premature Delivery in China- Systematic Review and Meta-Analysis
Source: Int J Public Health. 2023 Oct 9;68:1606226. doi: 10.3389/ijph.2023.1606226 (PMC10590883; doi:10.3389/ijph.2023.1606226)
Supplement: Supplementary file 1 [file DataSheet1.docx]

**Meta-analysis of the relationship between outdoor air pollutant exposure and preterm birth in China**

Xue Wang^a^*, Xin Wang^b^*, Chenghua Gao^a^, Xiaoqian Xu^a^, Lehui Li^a^, Yan Liu^a^, Zichao Li^a^, Yuan Xia ^a^, Xin Fang^a^

^a^ School of Public Health of Inner Mongolia Medical University, Hohhot, Inner Mongolia 010000, China

^b^ Division of Molecular Signaling, Department of the Advanced Biomedical Research, Interdisciplinary Graduate School of Medicine, University of Yamanashi, Chuo City 409-3898, Japan

*These authors are contributed equally to this work

*Corresponding Author*：Xin Fang Email: [18686066179@163.com](mailto:18686066179@163.com)

Journal: Environmental Science and Pollution Research





Fig.S1 Funnel plot of the association between exposure to PM10 and PTB. Pooled estimates of effect size are indicated by vertical points of diamonds, size of shaded area around the diamond is proportional to weight, and 95% CI are represented by horizontal line. (A, entire pregnancy; B, first trimester; C, second trimester; D, third trimester).





Fig.S2 Funnel plot of the association between exposure to SO_2_ and PTB. Pooled estimates of effect size are indicated by vertical points of diamonds, size of shaded area around the diamond is proportional to weight, and 95% CI are represented by horizontal line. (A, entire pregnancy; B, first trimester; C, second trimester; D, third trimester).





Fig.S3 Funnel plot of the association between exposure to NO_2_ and PTB. Pooled estimates of effect size are indicated by vertical points of diamonds, size of shaded area around the diamond is proportional to weight, and 95% CI are represented by horizontal line. (A, entire pregnancy; B, first trimester; C, second trimester; D, third trimester).





Fig.S4 Funnel plot of meta-analysis of the effect of different gestational exposures to PM_2.5_ on preterm birth in China (A, entire pregnancy; B, first trimester; C, second trimester; D, third trimester).


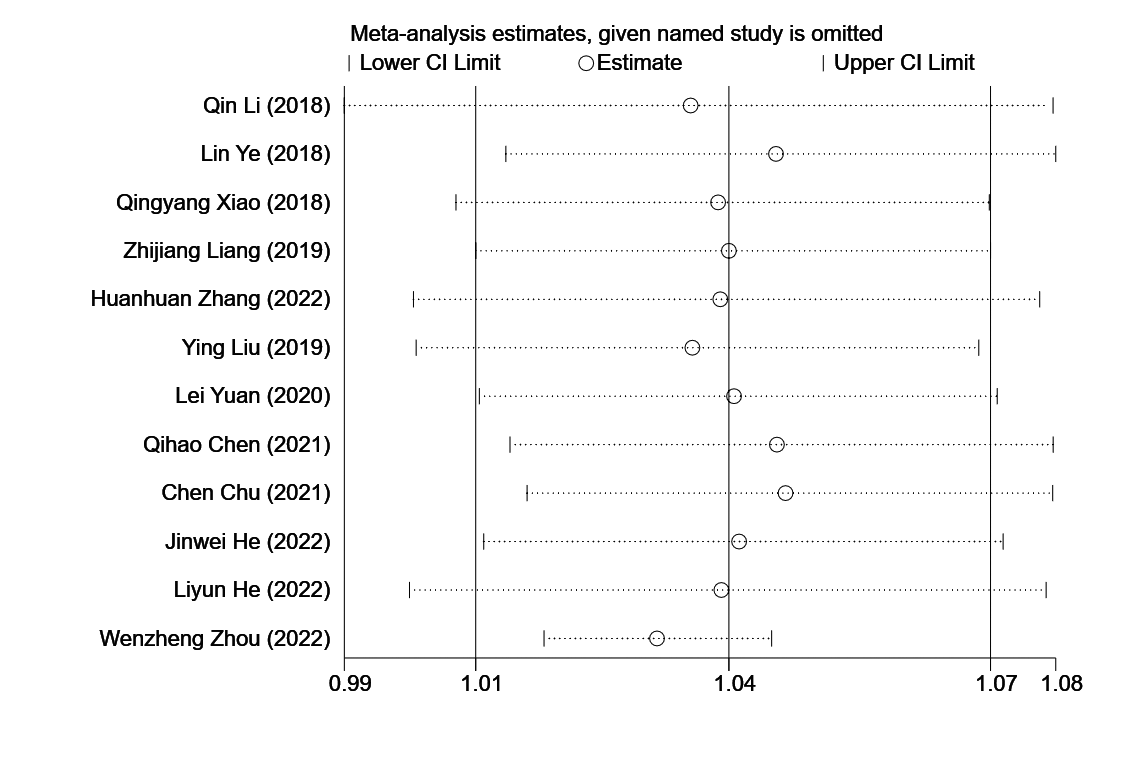


Fig.S5 Sensitivity analysis of the effect of PM_2.5_ exposure in entire pregnancy on premature delivery


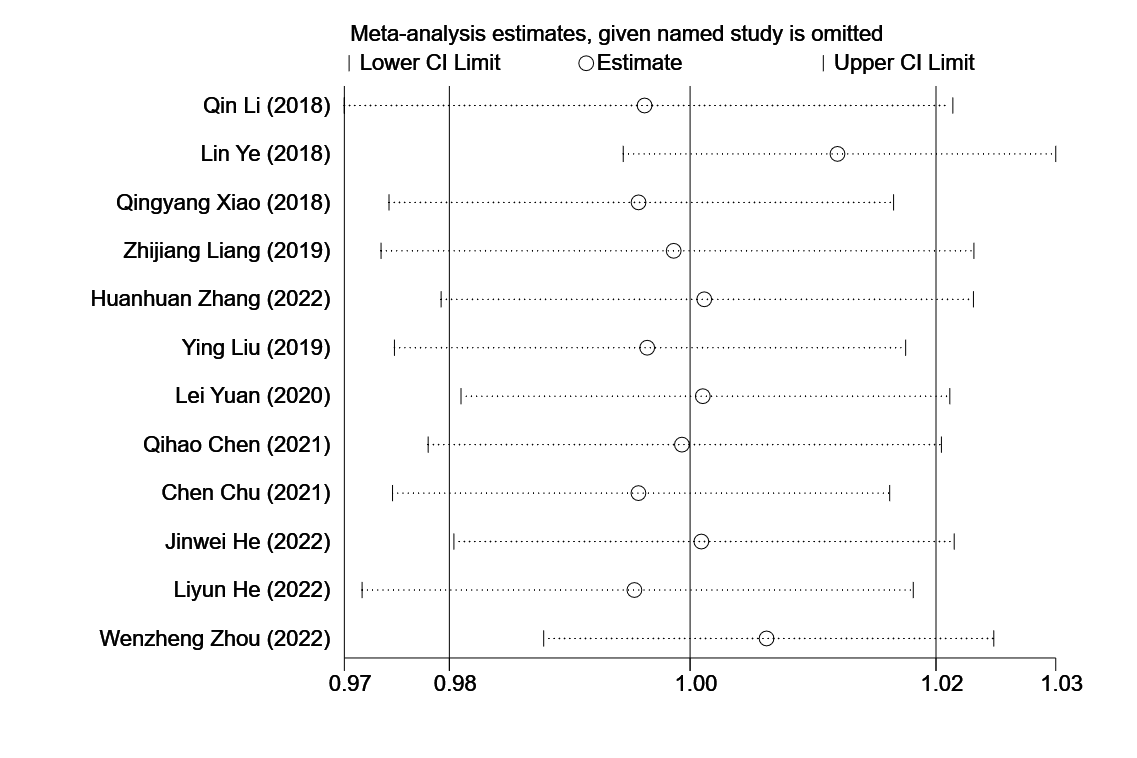


Fig.S6 Sensitivity analysis of the effect of PM_2.5_ exposure in the first trimester of pregnancy on premature delivery


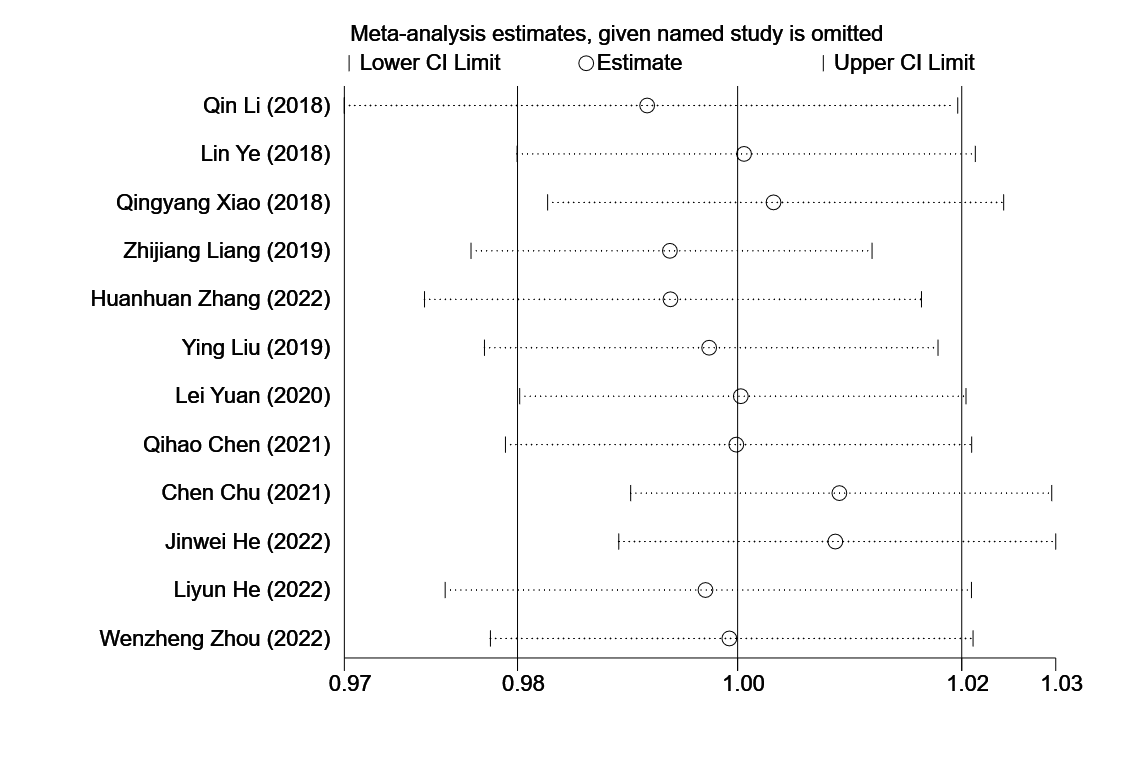


Fig.S7 Sensitivity analysis of the effect of PM2.5 exposure in the second trimester of pregnancy on premature delivery


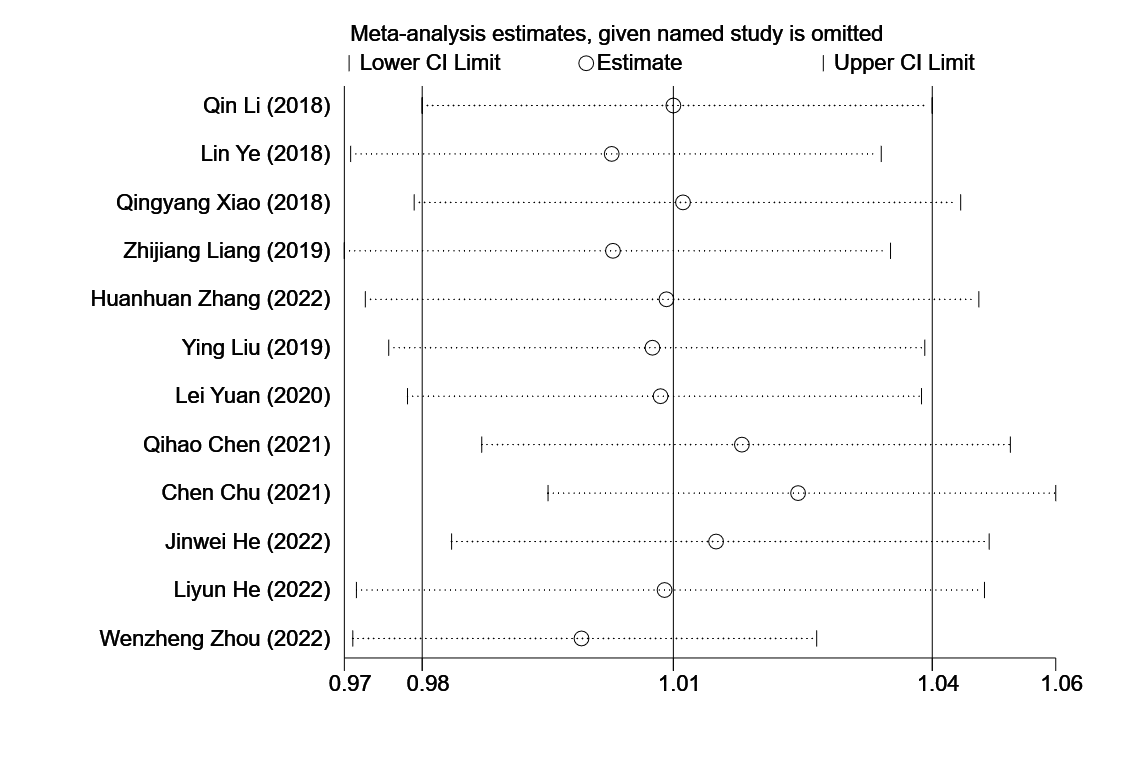


Fig.S8 Sensitivity analysis of the effect of PM2.5 exposure in the third trimester of pregnancy on premature delivery





Fig.S9 Funnel plot of meta-analysis of the effect of different gestational exposures to PM10 on preterm birth in China (A, entire pregnancy; B, first trimester; C, second trimester; D, third trimester).


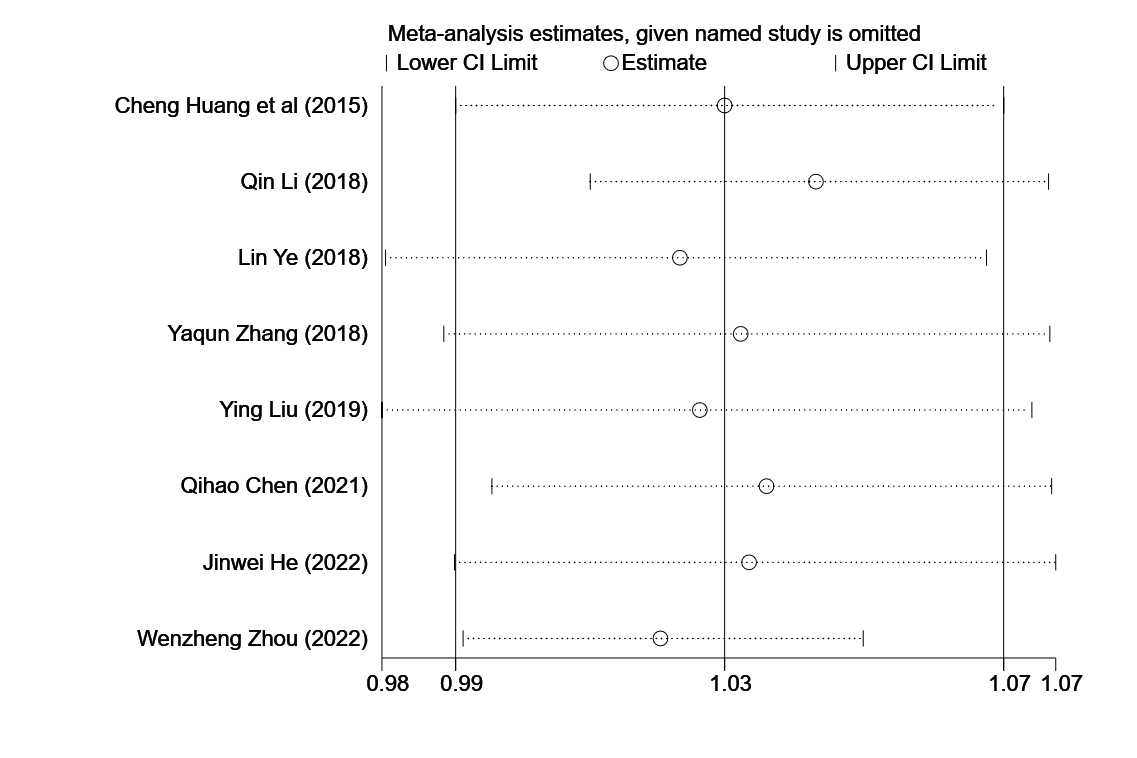


Fig.S10 Sensitivity analysis of the effect of PM10 exposure in entire pregnancy on premature delivery


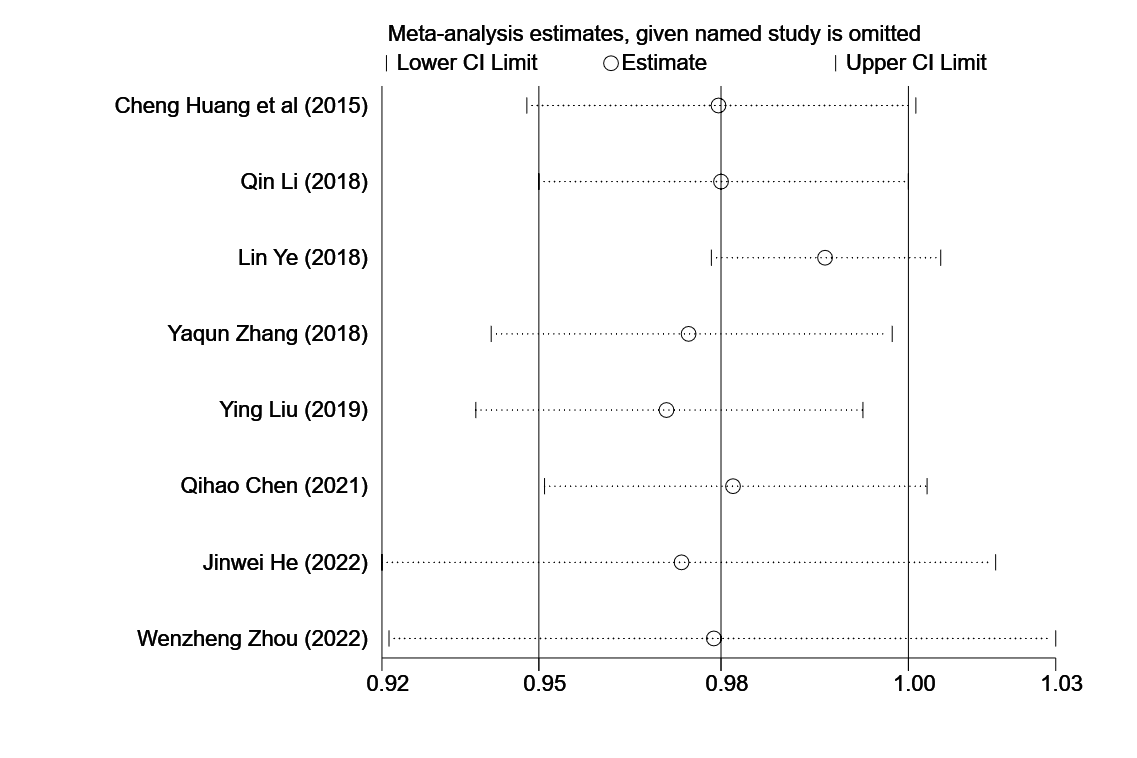


Fig.S11 Sensitivity analysis of the effect of PM10 exposure in the first trimester of pregnancy on premature delivery


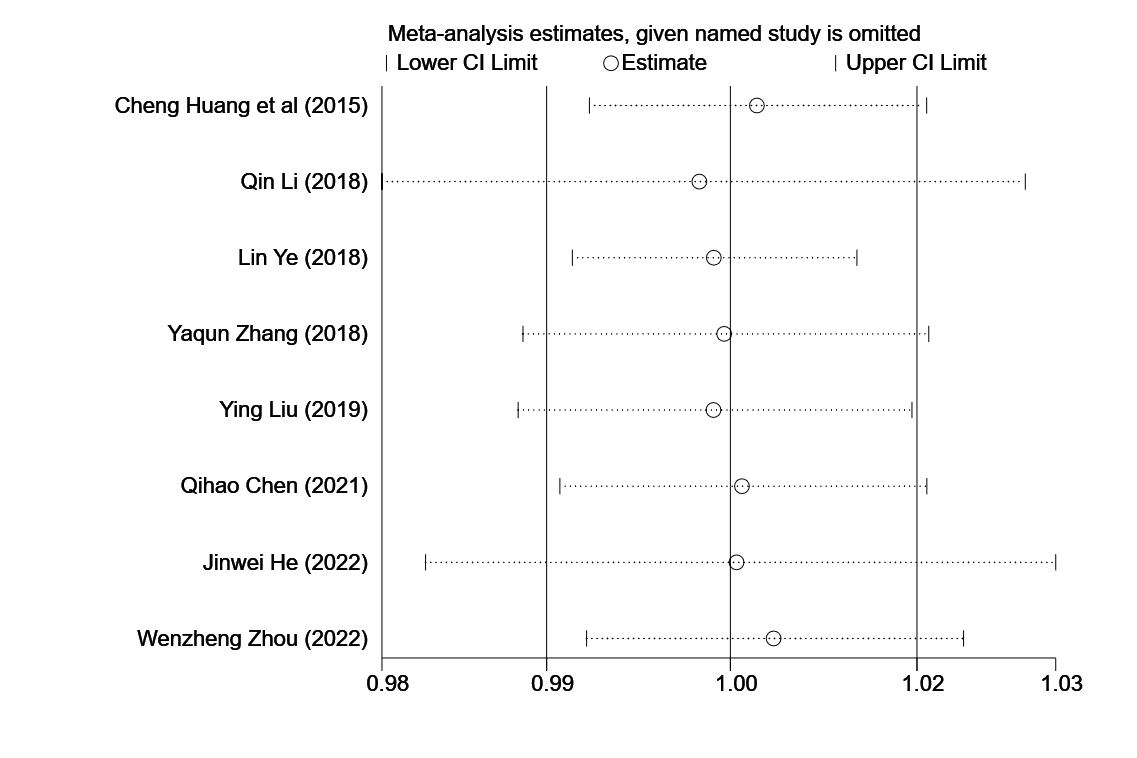


Fig.S12 Sensitivity analysis of the effect of PM10 exposure in the second trimester of pregnancy on premature delivery


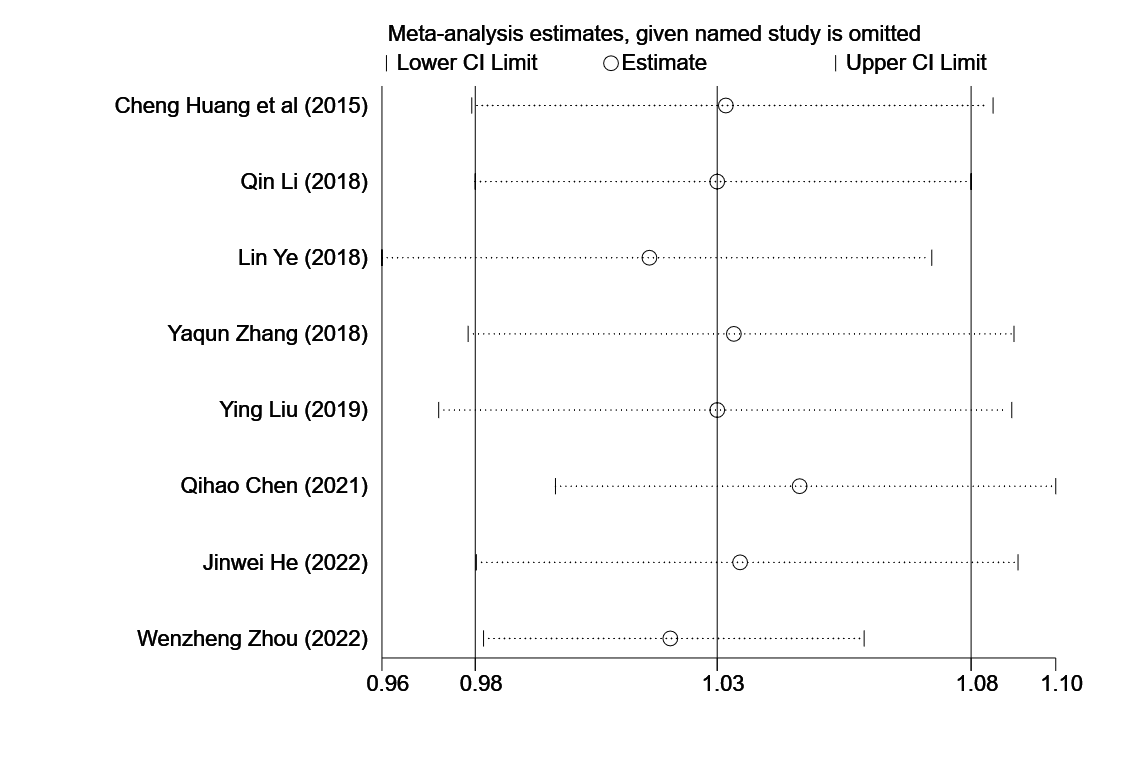


Fig.S13 Sensitivity analysis of the effect of PM_10_ exposure in the third trimester of pregnancy on premature delivery


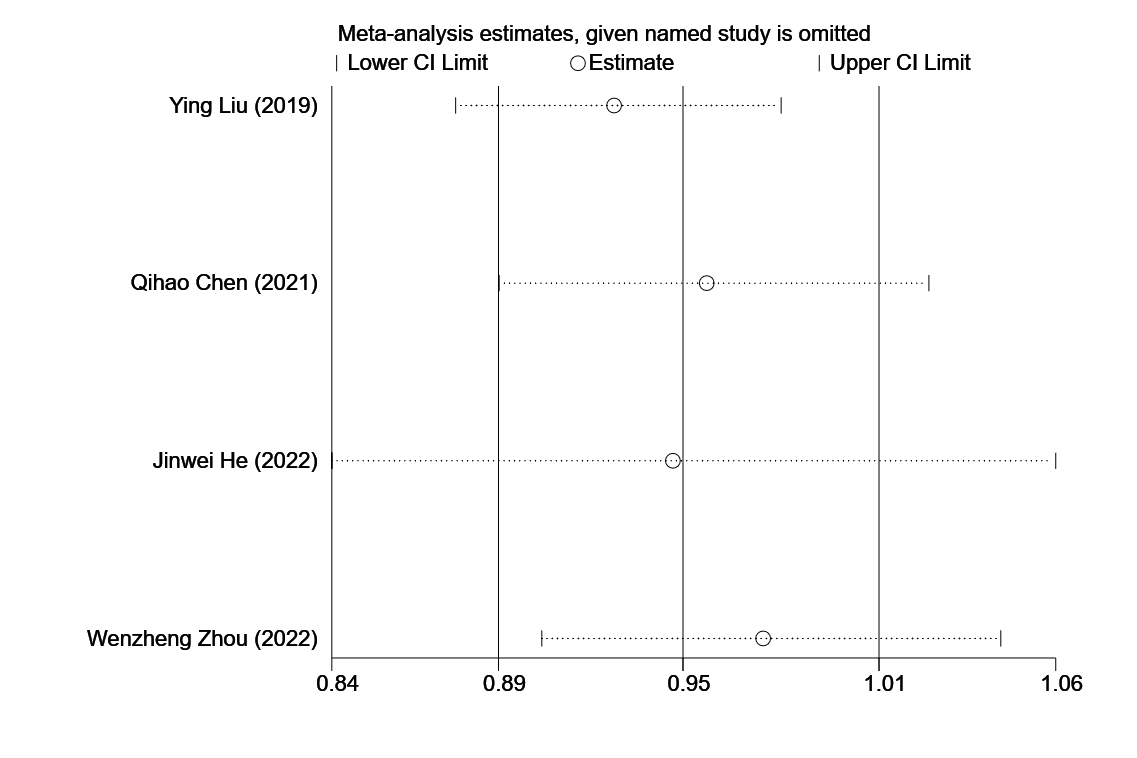


Fig.S14 Sensitivity analysis of the effect of SO_2_ exposure in entire pregnancy on premature delivery


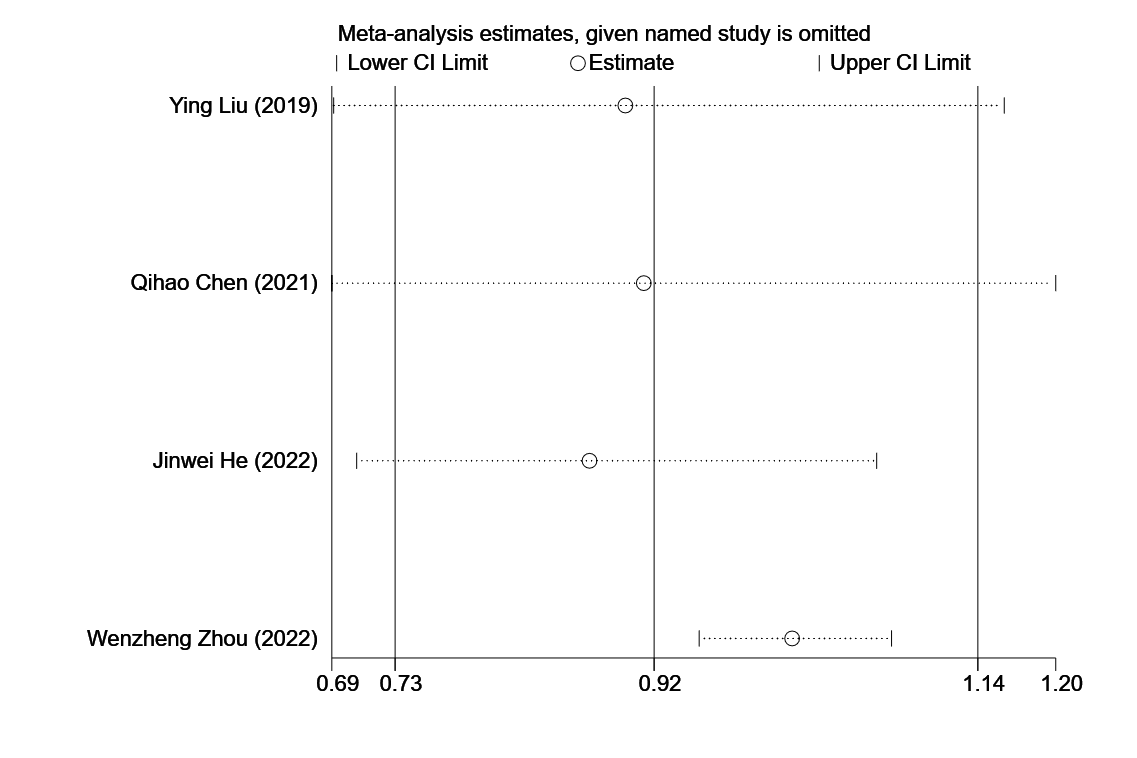


Fig.S15 Sensitivity analysis of the effect of SO_2_ exposure in the first trimester of pregnancy on premature delivery


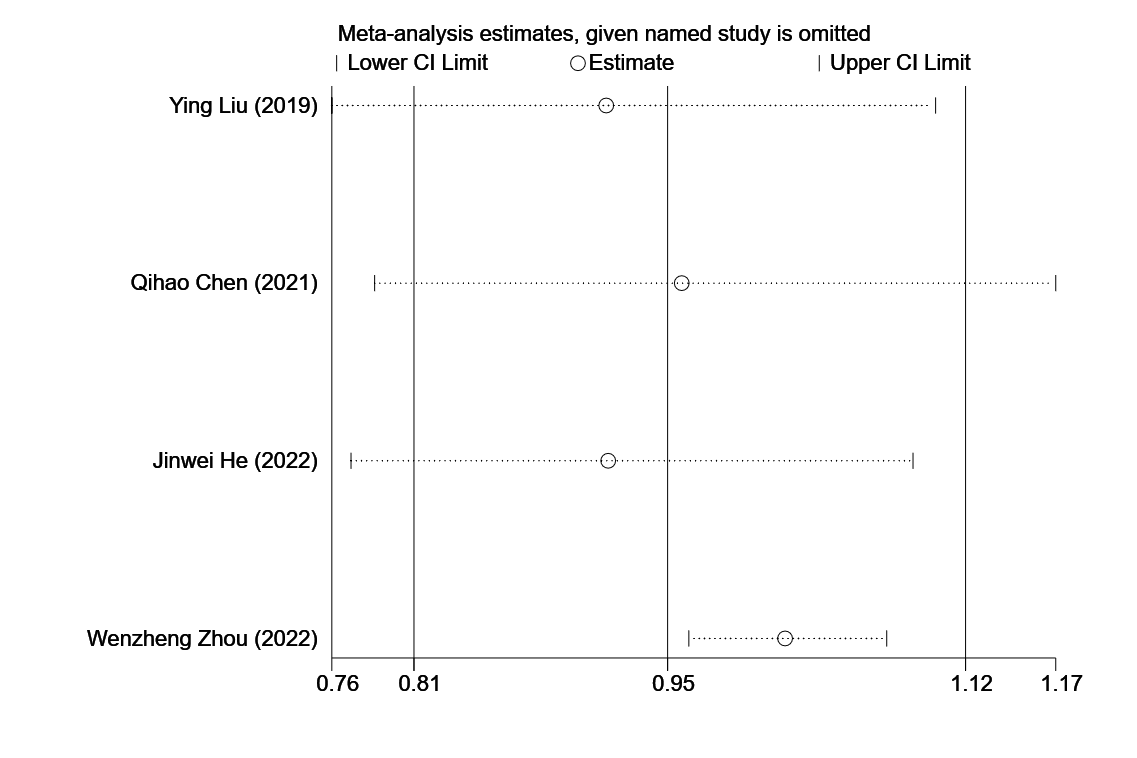


Fig.S16 Sensitivity analysis of the effect of SO_2_ exposure in the second trimester of pregnancy on premature delivery


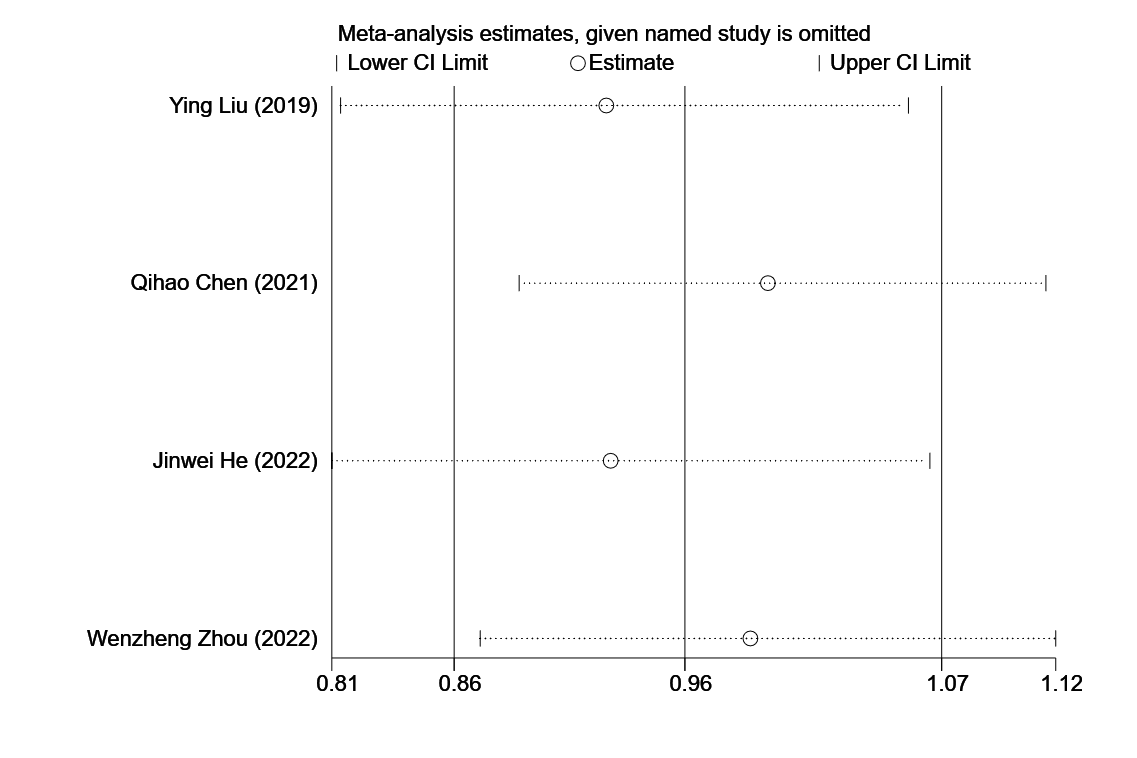


Fig.S17 Sensitivity analysis of the effect of SO_2_ exposure in the third trimester of pregnancy on premature delivery


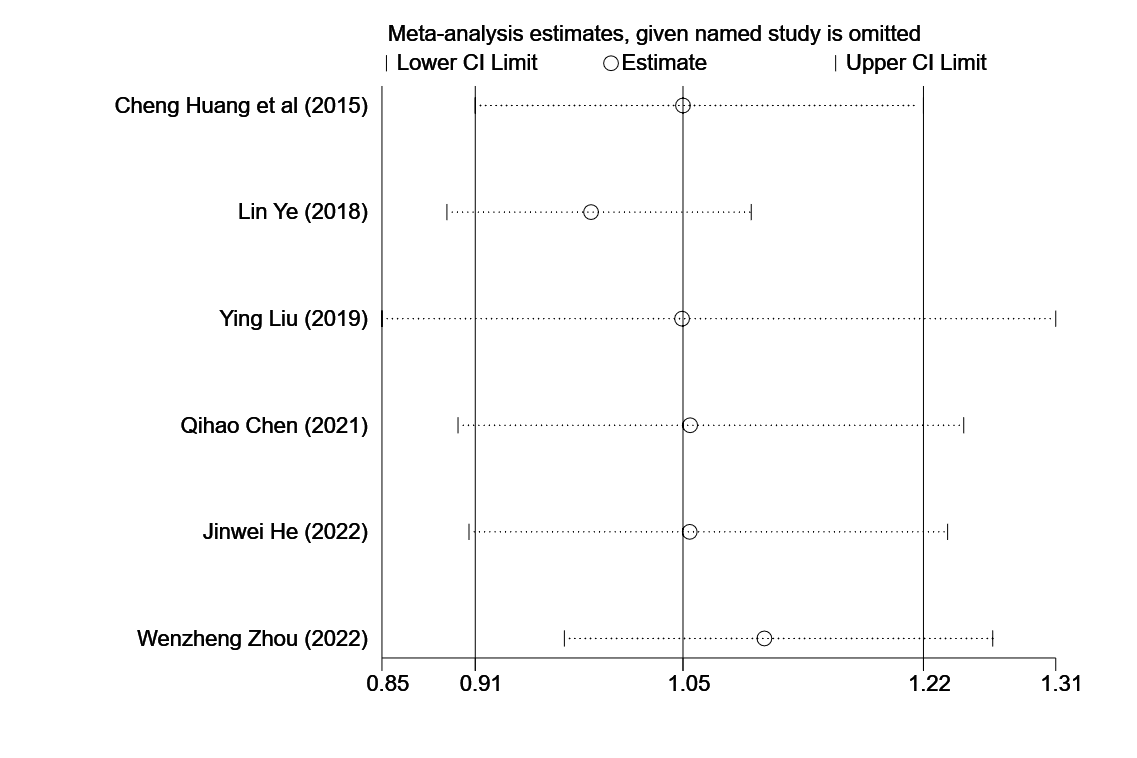


Fig.S18 Sensitivity analysis of the effect of NO2 exposure in entire pregnancy on premature delivery


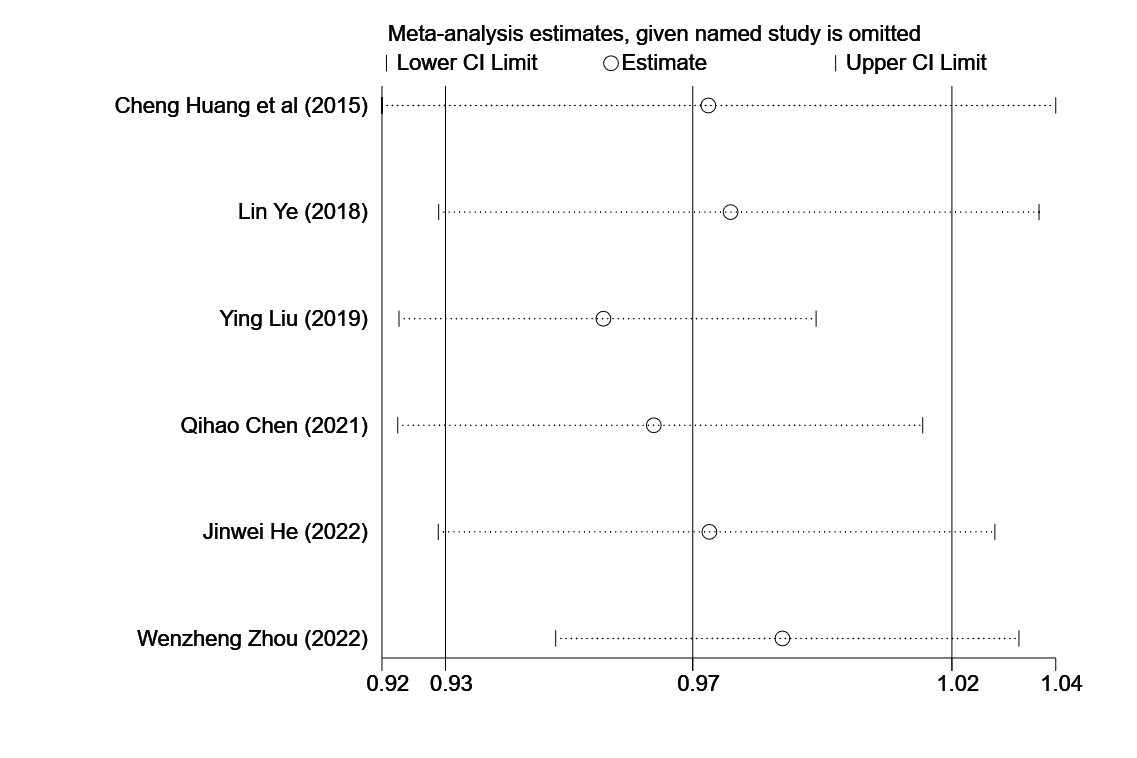


Fig.S19 Sensitivity analysis of the effect of NO2 exposure in the first trimester of pregnancy on premature delivery


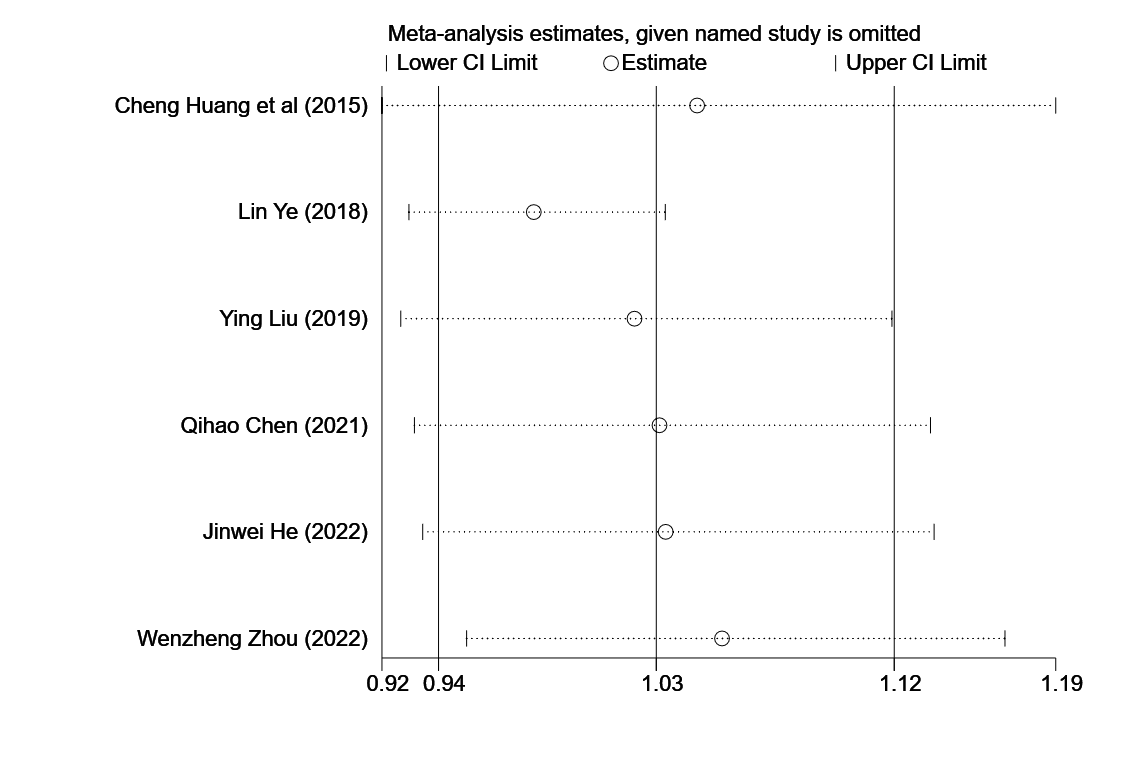


Fig.S20 Sensitivity analysis of the effect of NO2 exposure in the second trimester of pregnancy on premature delivery


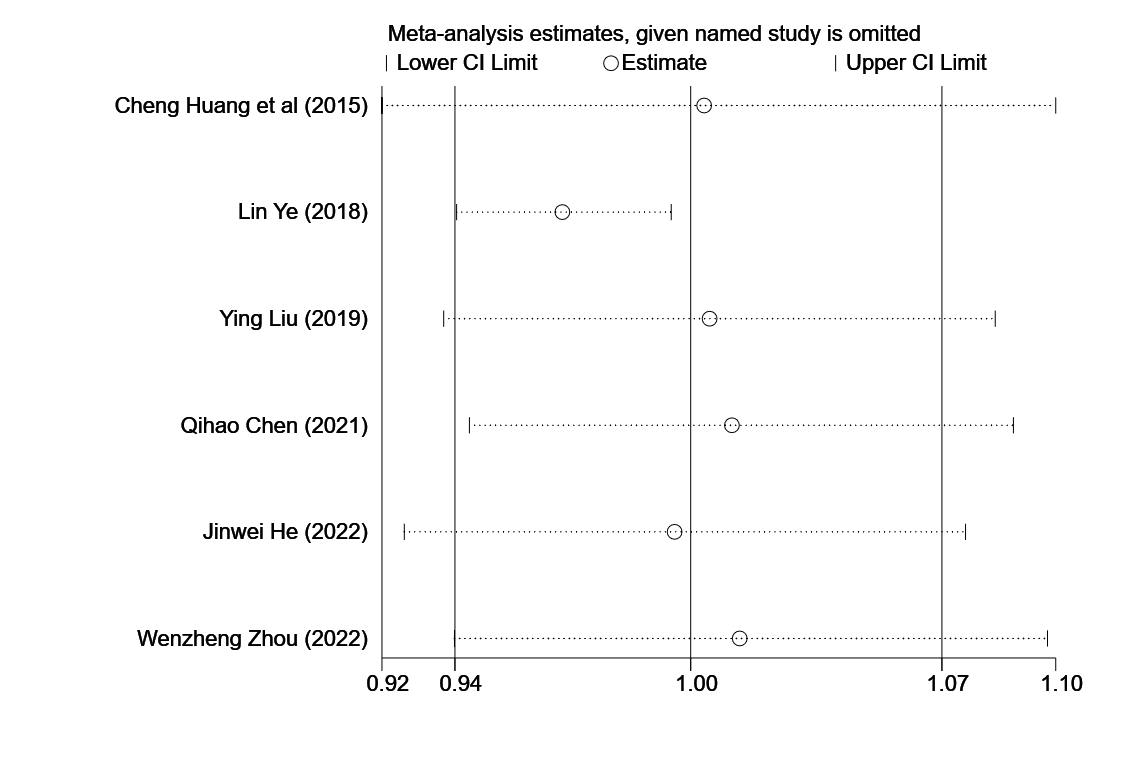


Fig.S21 Sensitivity analysis of the effect of NO_2_ exposure in the third trimester of pregnancy on premature delivery


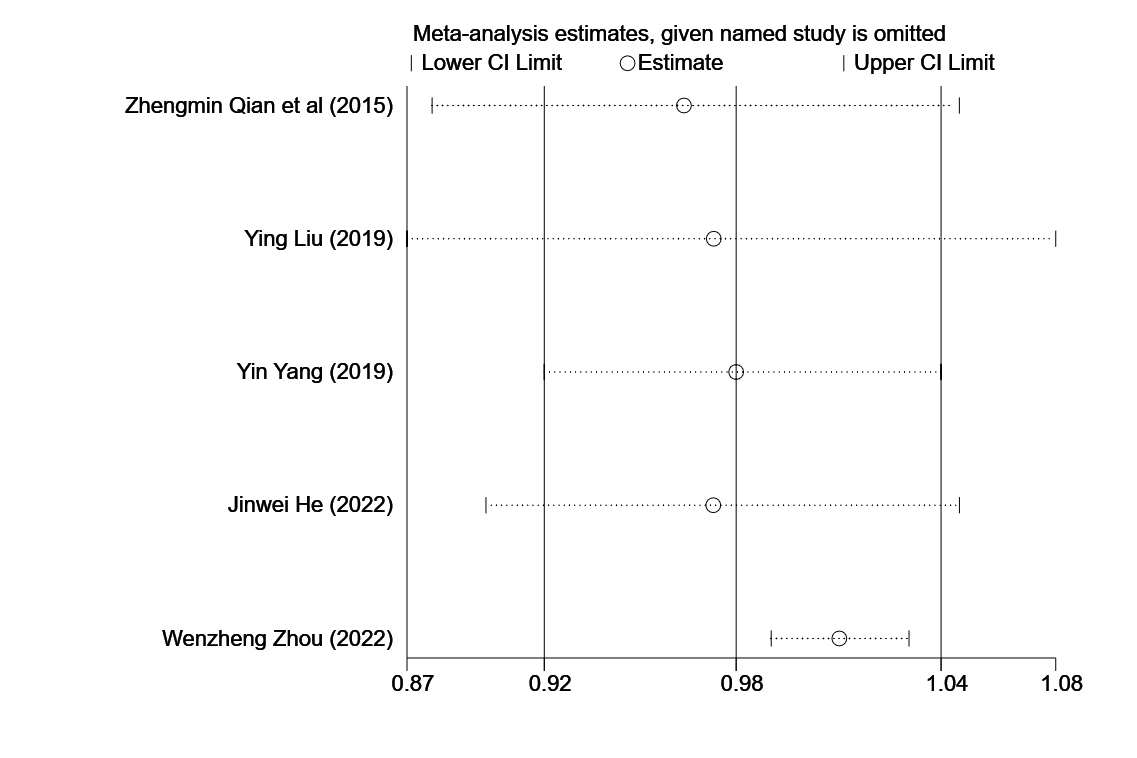


Fig.S22 Sensitivity analysis of the effect of O3 exposure in entire pregnancy on premature delivery


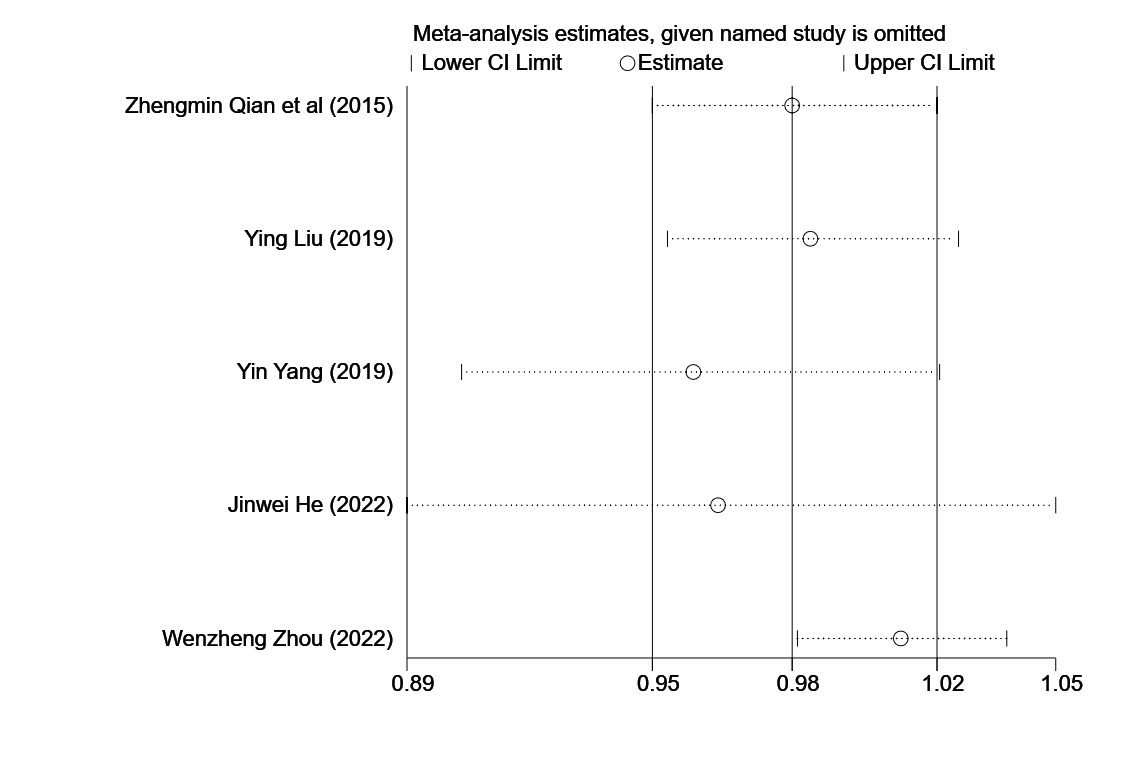


Fig.S23 Sensitivity analysis of the effect of O_3_ exposure in the first trimester of pregnancy on premature delivery


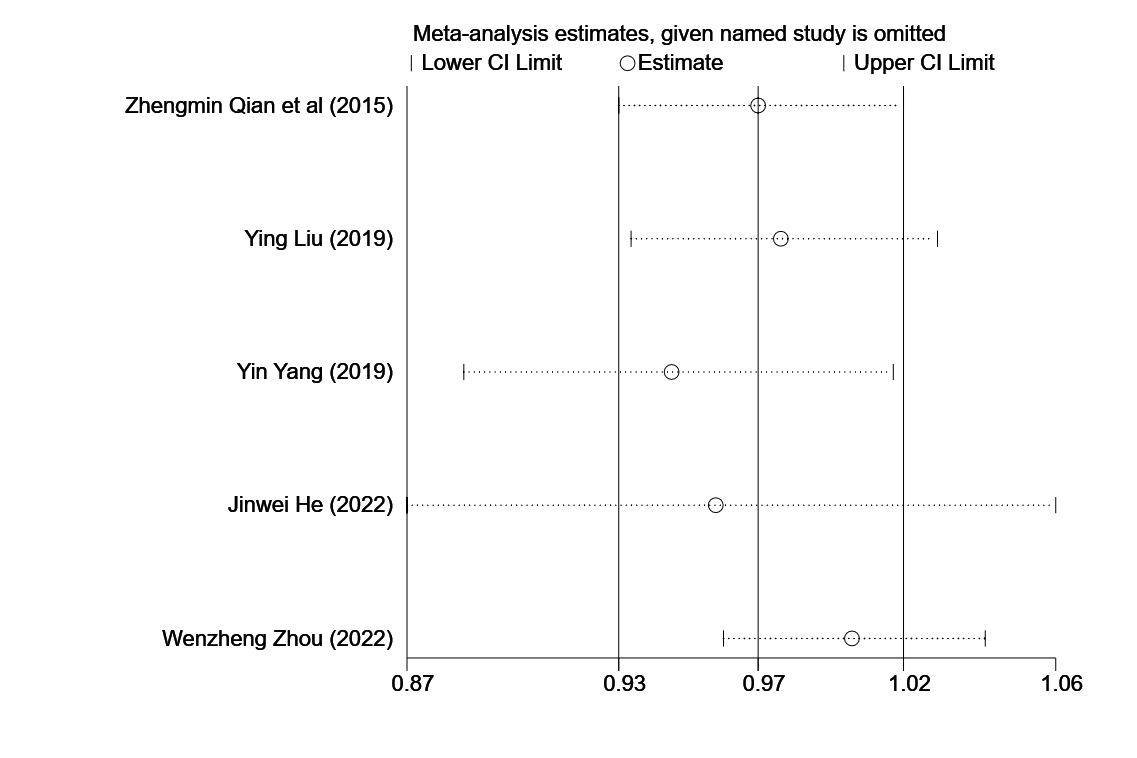


Fig.S24 Sensitivity analysis of the effect of O3 exposure in the second trimester of pregnancy on premature delivery


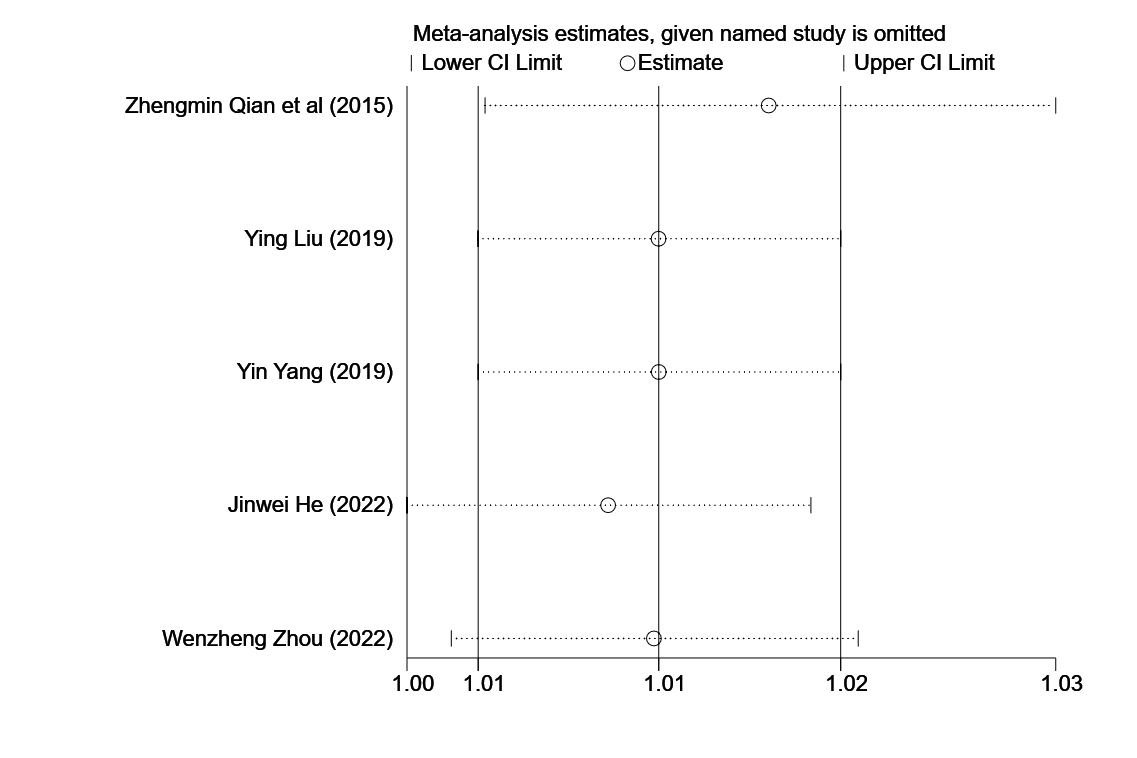


Fig.S25 Sensitivity analysis of the effect of O_3_ exposure in the third trimester of pregnancy on premature delivery
